# Describing the Breakbone Fever: IDODEN, an Ontology for Dengue Fever

**DOI:** 10.1371/journal.pntd.0003479

**Published:** 2015-02-03

**Authors:** Elvira Mitraka, Pantelis Topalis, Vicky Dritsou, Emmanuel Dialynas, Christos Louis

**Affiliations:** 1 Institute of Molecular Biology and Biotechnology, FORTH, Heraklion, Greece; 2 Department of Biology, University of Crete, Heraklion, Greece; 3 Centre of Functional Genomics, University of Perugia, Perugia, Italy; Genesis Laboratories, UNITED STATES

## Abstract

**Background:**

Ontologies represent powerful tools in information technology because they enhance interoperability and facilitate, among other things, the construction of optimized search engines. To address the need to expand the toolbox available for the control and prevention of vector-borne diseases we embarked on the construction of specific ontologies. We present here IDODEN, an ontology that describes dengue fever, one of the globally most important diseases that are transmitted by mosquitoes.

**Methodology/Principal Findings:**

We constructed IDODEN using open source software, and modeled it on IDOMAL, the malaria ontology developed previously. IDODEN covers all aspects of dengue fever, such as disease biology, epidemiology and clinical features. Moreover, it covers all facets of dengue entomology. IDODEN, which is freely available, can now be used for the annotation of dengue-related data and, in addition to its use for modeling, it can be utilized for the construction of other dedicated IT tools such as decision support systems.

**Conclusions/Significance:**

The availability of the dengue ontology will enable databases hosting dengue-associated data and decision-support systems for that disease to perform most efficiently and to link their own data to those stored in other independent repositories, in an architecture- and software-independent manner.

## Author Summary

The need for the construction of a dengue ontology arose through the fact that the incidence of dengue fever is on the rise across the world; the number of cases may be three to four times higher than the 100 million estimated by the WHO and a vaccine is still not available in spite of the significant efforts undertaken. Thus, control of dengue fever still relies mostly on controlling its mosquito vectors. Large amounts of entomological, epidemiological and clinical data are generated; these need to be efficiently organized in order to further our comprehension of the disease and its control. IDODEN aims to cover the different aspects and intricacies of dengue fever and syndromes caused by dengue virus(es). It contains more than 5000 terms describing epidemiological data, vaccine development, clinical features, the disease course, and more. We show here that it can be a helpful tool for researchers and that, in addition to allowing sophisticated search strategies, it is also useful for tasks such as modeling.

## Introduction

Dengue fever is a viral vector-borne disease, limiting the spread of which relies, directly or indirectly, on the control of the vectors that transmit it [1]. The present lack of vaccines, despite the substantial effort invested for their development, combined with the global increase of dengue cases over the last decades and the fact that a reversion of this trend is not yet apparent [2] make it crucial to identify and mobilize resources in the domain of disease prevention and management, which are either novel, or have not yet been fully established. Although several modern techniques such as GPS-assisted Geographic Information Systems (GIS) [3–7] and decision support systems [8] have found their way into the collection of tools against dengue, the full deployment of Information Technology (IT)-related applications is still lagging behind. Given the spread of the disease across all continents with the exception of Antarctica, it is necessary to improve strategies, to streamline and manage data availability and to enable their access across international boundaries and different technical platforms.

Usage of ontologies was only recently adopted to act as specific IT tools in the field of life sciences. This methodology has been shown to be exploited for efficient searches of databases, for modeling and, if widely adopted by a relevant research community, to achieve enhanced interoperability of IT resources. In computer science ontologies represent a formal, explicit specification of a conceptualization and provide a common vocabulary describing classes (or terms or sets or concepts) and attributes (or properties or relationships or relations) in a given domain. The complete definitions, listing of synonyms, additional information pertaining to terms (e.g. cross-references) and in particular the usage of defined relations linking terms, renders the use of ontologies the tool of choice for driving complicated databases. Searches that have been directed to use these features of ontologies can perform much more efficiently and, if the same ontologies are in different databases, there is the additional advantage of having the capability of searching across different platforms, thus achieving enhanced interoperability. Finally, ontologies can be used to model complicated biological knowledge [9–10].

An ontology, in information sciences, provides definitions of terms in a given domain, as well as, most importantly, the relations that link these terms to each other. Based on the relationships between terms, the parent-children configuration leads to a tree-like format when an ontology is laid out graphically. The mandatory integration of relations in ontologies differentiates them from simple controlled vocabularies and renders them powerful. The first bio-ontology, the Gene Ontology (GO), published 14 years ago, was presented as a tool to be used towards the “unification of biology” [11, 12]. In the time that followed, the GO has grown and developed and is now used by researchers from many different fields: indeed, genomics cannot nowadays be imagined without the GO.

Not only has the GO been crucial in the annotation of genes, genomes and related experiments [13–15], it has also, in turn, provided the impetus for the development of additional IT tools that have greatly enhanced the analysis of the data resulting from high-throughput research. For example, specific database searches have obtained an additional dimension; it is now possible to search for common attributes (e.g. cellular localization, functions, and so on) in repositories that store data from different organisms, obtained by diverse methodologies and by a variety of researchers, and determine functional kinships between gene products that were previously hidden. This interoperability of databases may indeed represent one of the most efficient usages of ontologies, and of the GO specifically [16]. In the 14 years since the publication of the GO, tens of novel bio-ontologies were constructed and were deposited in repositories such as the OBO Foundry [17] or the National Center for Biomedical Ontology [18], describing a wide variety of domains such as anatomy [19], brucellosis [20], chemical entities of biochemical importance [21] and others. Nevertheless, although in general most researchers will agree *a priori* on the importance of ontologies as tools, it is mostly the field of informatics that has started making extensive use of them while, with the exception of the GO, ontologies are still “underused” in the life sciences domain. Efforts to surpass methodological difficulties in specifying or understanding and, therefore using, ontologies are underway, especially in health sciences [22, 23].

Within the framework of the VectorBase project [24] we initiated the development of a set of ontologies that can be used to describe arthropod vectors and the diseases they transmit [25]. These ontologies deal with the anatomy of mosquitoes and ticks [26], mosquito insecticide resistance [27] and malaria [28]. Here we describe the latest addition to this set of IT tools, namely the ontology for dengue, called IDODEN. We emphasize the potential usage of the IDODEN, rather than its contents and its architecture, in order to promote a better understanding and appreciation of ontologies by the dengue research community and other life scientists. Finally, we provide examples of how the ontology can be used in order to model dengue-specific use cases.

## Methods

Historically, the early bio-medical ontologies were structured according to the OBO Flat File Format [29], which is now superseded by the Web Ontology Language (OWL) specifications [30] conforming to the recommendations of the World Wide Web Consortium (W3C) as the standard for developing ontologies across different domains of research. We therefore wrote IDODEN in OWL, although an OBO version is available, generated using the OWLtoOBO software [31]. Protégé [32], an open-source ontology editor was used for the construction of IDODEN. In this paper terms are italicized and enclosed in quotation marks, relations are italicized and definitions of terms are enclosed in quotation marks.

### Strategy of the development of IDODEN

We decided to use the same architecture, originally chosen for IDOMAL, for the construction of IDODEN. The main reason for that was the fact that we had decided to construct vector-borne disease ontologies as extensions to IDO, the Infectious Disease Ontology [33]. The IDO project is a loose collaborative undertaking that links together ontology developers interested in a variety of infectious diseases. We have to state here that in spite of our efforts, a few discrepancies between IDO and IDOMAL still exist [34], and these are carried into IDODEN. We are hopeful to eliminate these differences in the near future. Therefore, IDODEN still uses the Basic Formal Ontology (BFO) [35, 36] as a small, upper level ontology, something that helps the exchange of pertinent information with other related ontologies that also use BFO at the upper level. The BFO does not contain terms that are specific to a given scientific domain, It should be stated here, that we have not yet decided whether to “upgrade” the vector-borne-disease ontologies to BFO 2.0 [37] awaiting its “formal” adoption by the ontology development community. Should such a decision be taken in the future, again the fact that our ontologies have a similar blueprint would render this operation easier.

There is one requirement for ontologies to become really useful tools for the community for which they are intended, namely that they be widely accepted and subsequently used/adopted by the community. Additionally, to promote interoperability wherever terms/concepts are not “thematically” part of the domain described by the ontology in question (e.g. dengue for IDODEN) these should be imported directly from ontologies that are themselves used/adopted by a large number of communities. While the first of these requirements fully depends on the free availability of the ontology as well as, importantly, its objective quality, the second one relies on abiding by rules such as, for example, the ones set by the OBO Foundry [17]. Among others, the OBO Foundry principles require availability as open source as a development model, shared syntax (OBO or OWL), a clearly specified and clearly delineated content and unambiguous definitions of relations, We decided early on to have our ontologies follow the OBO Foundry rules in order for them to provide the highest degree of “cross-talk” between them and other ontologies. This prospect was assisted by importing as many terms as possible from other ontologies, rather than describing them *de novo*. In other words, these terms have the same ID and the same distinctive features in a given ontology (here, IDODEN) as in the one they were imported from. Thus, definitions, parenthood relations, and synonyms remain the same; this approach is known as MIREOT: the Minimum Information to Reference an External Ontology Term [38]. As already stated, we also decided to base the architecture of the IDODEN on that of the IDOMAL. This has some distinct advantages: i) should all vector-borne disease ontologies that we construct have the same architecture, it would be easier to use a common matrix for their development; moreover, this would make it simpler, if so decided in the future, to join these ontologies into a major single one covering the domains of more than one disease; ii) a second advantage of this is that should these ontologies be used to drive specific databases or other IT tools such as decision support systems, the similar pattern followed by them would enable the database engineers to incorporate the ontologies in question directly into their database and use them for specific purposes (e.g. searches); and iii) thirdly, when our ontologies incorporate IDO immediately below BFO, and at the top of the specifics of “our” diseases, any potential change required will only have to be “designed” once to be adapted for all ontologies describing vector-borne diseases.

Finally, in order for IDODEN to better describe the complexity of vector-borne diseases we decided to have the term “host” describe the human host/patient throughout the ontology while, obviously, the mosquito is always referred to as “vector”.

IDODEN is freely available for browsing and downloading in the OWL format at the NCBO Bioportal (http://bioportal.bioontology.org/ontologies/3174) while we can make available the OBO-format based version on request.

## Results and Discussion

### IDODEN: The architecture

The current version of IDODEN (version 1.0) contains 5035 terms, of which 1482 are imported and 3553 were newly created for this ontology. The imported terms stem from a variety of other ontologies, which are recognized as authoritative by the community. Not unexpectedly, given the approach chosen (as mentioned above, the overall format of IDODEN is that of its malaria counterpart), almost half of the imported terms have been taken from IDOMAL, while ChEBI [21], GO [11, 12], MIRO [27] and the Environment Ontology ENVO [39] follow in that order. A list of all donor ontologies can be found in Table 1. A large number of the newly-created terms represent the different dengue virus strains, which have been identified by the dengue research community. Although the inclusion of such instances in an ontology is not necessarily customary it is useful here since IDODEN was constructed with the annotation of entries in databases in mind. In this context, we have also taken care to keep IDODEN updated since the release of its alpha version, which was made available in November 2012. Thus, for example, the current version of the ontology (version 1.0) also contains DENV-5, the latest “addition” to the dengue virus serotype set [40]. A regular cycle for updating has not been fixed, but every change incorporated in IDODEN, usually depending on community input or literature-dependent changes/additions are immediately moved into the latest version located at the NCBO Bioportal.

**Table 1:** List of the ontologies and controlled vocabularies from which terms were borrowed and imported into IDODEN, as well as the number of these terms.

**Ontology**	**Number of terms**
IDOMAL—Malaria Ontology	721
CHEBI—Chemical Entities of Biological Interest Ontology	251
GO—Gene Ontology	115
MIRO—Mosquito Insecticide Resistance Ontology	99
ENVO—Environment Ontology	63
SYMP—Symptom Ontology	42
NCBITaxon—National Center for Biotechnology Information (NCBI) Organismal Classification	39
CL—Cell Ontology	34
BFO—Basic Formal Ontology	29
PR—Protein Ontology	26
IDO—Infectious Disease Ontology	26
IAO—Information Artifact Ontology	7
SO—Sequence Ontology	7
OBI—Ontology for Biomedical Investigations	5
VO—Vaccine Ontology	5
OGMS—Ontology for General Medical Science	4
UBERON—Uber Anatomy Ontology	2
CARO—Common Anatomy Reference Ontology	1
EFO—Experimental Factor Ontology	1
FB-BT—Drosophila Gross Anatomy Ontology	1
FLU—Influenza Ontology	1
GRO—Gene Regulation Ontology	1
HP—Human Phenotype Ontology	1
NPO—NanoParticle Ontology	1
IDODEN—Dengue Ontology	3553

All of these ontologies and controlled vocabularies are available through the NCBO Bioportal (http://bioportal.bioontology.org/ontologies).

IDODEN presently uses 12 relations (Table 2. These have been imported from the Relations Ontology [41] and other collections of relations that are listed by the OBO Foundry [17]. As is often the case, some relations are not intuitively easy to understand, but become clear when placed in context as in the examples in Table 2.

**Table 2:** The Table lists all relations used in IDODEN.

**relation**	**example**
*is_a*	*“dengue fever”* SubClassOf *is_a* some *“infectious disease”*
*agent_in*	*“Aedes albopictus”* SubClassOf *agent_in* some *“dengue transmission”*
*bearer_of*	*“Aedes albopictus”* SubClassOf *bearer_of* some *“Dengue virus”*
*happens_during*	“*ascites”* SubClassOf *happens_during* some *“clinical manifestation of dengue”*
*has_role*	*“Aedes albopictus”* SubClassOf *has_role* some “*infectious agent vector role”*
*inheres_in*	*“dengue virus seroprevalence”* SubClassOf *inheres_in* some “*human population”*
*part_of*	*“acquired immunity to dengue”* SubClassOf *part_of* some “*immunity”*
*participates_in*	*“dengue C protein”* SubClassOf *participates_in* some *“dengue virion assembly”*
*preceded_by*	*“vitellogenic stage”* SubClassOf *preceded_by* some *“previtellogenic development”*
*realized_by*	*“response to visual cue”* SubClassOf *realized_by* some *“adult vision”*
*realizes*	*“progression of dengue fever”* SubClassOf *realizes* some *“dengue shock syndrome”*
*results_in*	*“asymptomatic dengue”* SubClassOf *results_in* some “*convalescence”*

One randomly picked example is listed for each one of them.

The relation “*located_in*”, used in Fig. 8 is used by the GAZ ontology and not IDODEN.

Being based on BFO as a top-level ontology, the two uppermost classes of IDODEN are “*continuant*” and *“occurent”*. The latter contains all processes, developmental stages and disease-related temporal regions. “*process*” contains all processes, independent of whether these are related to, for example, epidemiology, control, diagnosis and therapy or biological processes of vector, host and pathogen. The “*continuant*” class is by far the largest and most heterogeneous class of IDODEN. We should caution here the “lay” reader of the ontology that in several cases it is not immediately apparent why two terms that describe entities that at first glance are very different from each other, are listed as children of the same parent. For example, the “*Breteau index*” [42] is a sibling of “*parous rate*”, simply because both are children of “*measurement datum*”. Needless to say that when the ontology is used by an IT tool, these apparent paradoxes have no negative effect whatsoever on the usage of the ontology.

Given the close kinship between IDODEN and IDOMAL, and in accordance with the latter, the dengue ontology regularly separates specific classes in the hierarchies of knowledge as members of distinct groupings (see Table 3): dengue fever, i.e. the actual disease, dengue vector, dengue host, and dengue virus, i.e. pathogen. The populations of the latter, where appropriate, are also handled separately from individuals. In addition, another related category, “multi-organism” is used to define all processes that relate to more than one of the above three such as, for example, vector-pathogen interactions. This separation leads to an overall reduction of the number of individual terms included in the ontology without a loss of information. The separation into these kinds of groupings also proved convenient in terms of a necessary modification: with the recent discovery of the 5th DENV serotype [40] only a few terms had to be added rather than adding a multitude of serotype-specific terms.

**Table 3:** Definitions of the key players of dengue fever.

**Ontology term**	**Definition**
dengue host	The host of a dengue virus
dengue virus	The assembled virus of dengue
dengue vector	A mosquito of the genus Aedes transmitting dengue
dengue fever	An infectious disease caused by the dengue virus
disease vector population	A given population of dengue vectors in a specific area
human population	A collection of human beings
multi-organism process	Any process by which an organism has an effect on another organism of the same or different species

An example of the advantage offered by the groupings can be seen in the hierarchy of the term “*quality of vector*”. The term “*anthropophily*”, defined as “The preference for feeding on humans” is common to all vectors of the disease. No need, therefore, to include additional terms such as “*anthropophily of Aedes aegypti”*, “*anthropophily of Aedes albopictus”*, and “*anthropophily of Aedes polynesiensis”*. These vectors are described, elsewhere in IDODEN, as the hierarchically last children of the series “*object->biotic object-> … ->vector organism->Aedes->Aedes nnnnnn”*, whereby “*nnnnn*” stands here for each individual species that has the “*dengue vector*” role. A potential database item could then be annotated with both the terms “*anthropophily*” and the actual species name, thus reducing the number of terms in the ontology without loosing specificity.

### IDODEN: The contents

**Figure 1:**
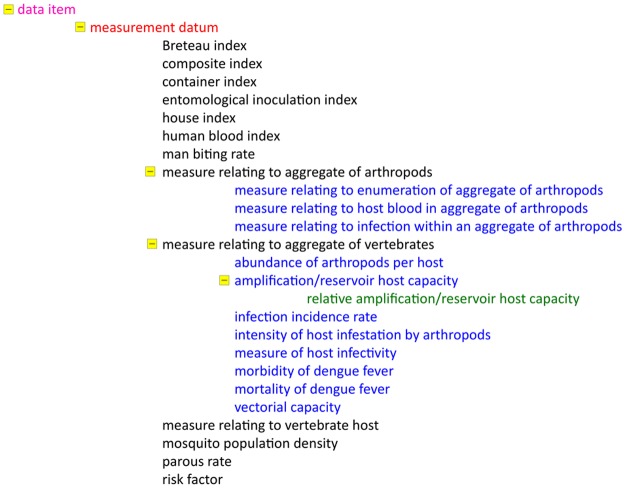
The *“data item*” hierarchy of terms in IDODEN. Font colours indicate the different levels of the hierarchy while a small yellow box in front of a term shows that it has children. The minus sign indicates that all child terms are listed.

A few examples will illustrate the contents of IDODEN, which, due to the high number of terms included in it, cannot be presented here in detail. We chose examples, which are “unique” to the dengue ontology, even if some of them, such as the first example presented, are of a more general nature; it deals with the hierarchy of the term “*data item*”, which belongs to the general term “*information content entity*” (Fig. 1). It should be noted here that in all figures in which hierarchies are shown, grouped terms appear alphabetically, the way they are listed in the actual ontology. In IDODEN, like in many application ontologies, several terms are omitted in some hierarchies; the best example of this is with taxonomic ranks. In cases that data need to be annotated in a database or a decision support system (DSS) with such a missing term, a term higher up in the hierarchy is used instead. To maximize the usage of ontological terms we nevertheless did include the “*information content entity*” hierarchy and expanded it to cover certain needs; under “*measurement datum*” we find a series of indexes, most of which are used in epidemiological and entomological studies. Examples of the latter are “*Breteau index*”, *i.e.* the number of containers that contained larvae for every 100 houses surveyed, and the “*house index*”, that refers to the number of houses in which *Aedes* larvae and/or pupae were present. Of course terms such as “*man biting rate*” and “*mosquito population density*” are also found here.

**Figure 2:**
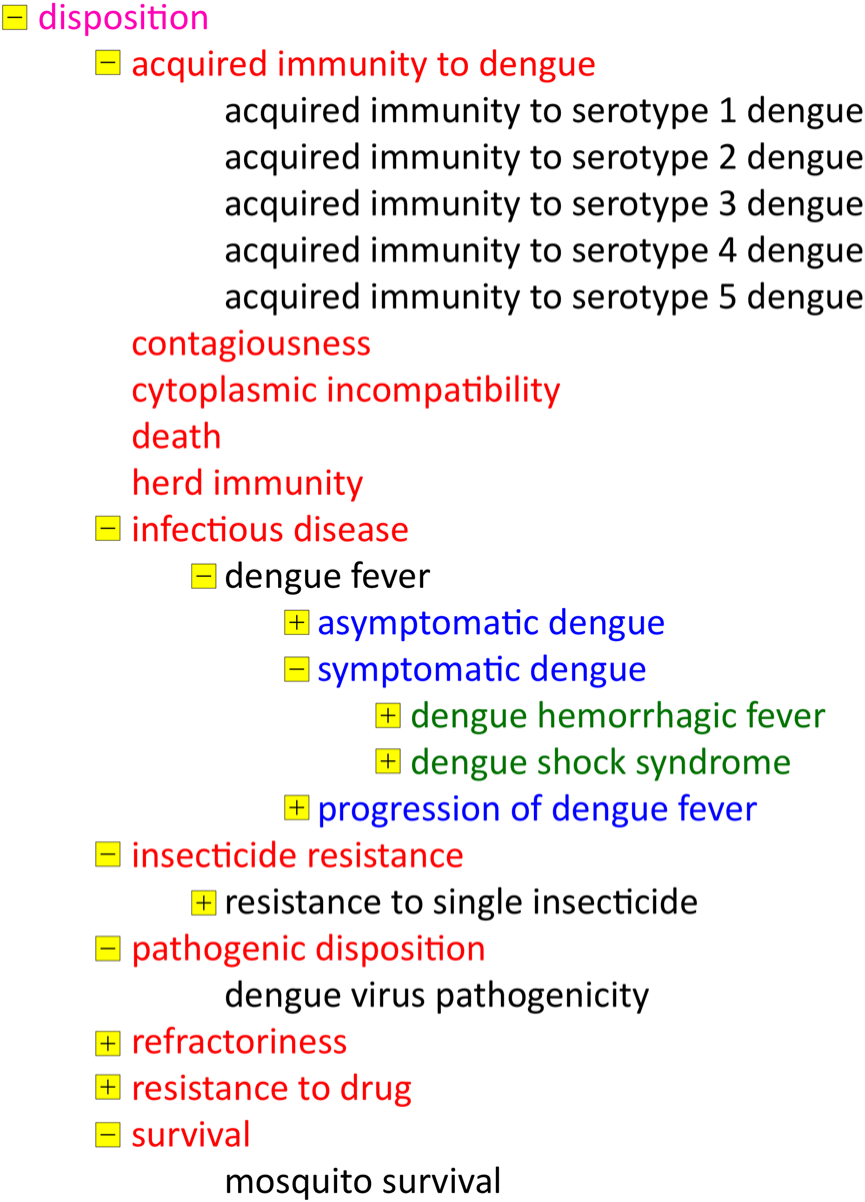
The *“disposition*” hierarchy of terms in IDODEN. Font colours indicate the different levels of the hierarchy while a small yellow box in front of a term shows that it has children. The minus sign indicates that all child terms are listed while the plus sign indicates the presence of child terms that are not shown in the Figure.

The second example of the contents displays the hierarchy “*disposition*”, a term that often leads to misunderstandings. In BFO, the definition of “*disposition*” is “A realizable entity that essentially causes a specific process or transformation in the object in which it inheres, under specific circumstances and in conjunction with the laws of nature” [35, 36]. A general formula for dispositions is: X (object) has the disposition D to (transform, initiate a process) R under conditions C” [43]. This definition best explains why infectious diseases and, in the particular case of IDODEN, dengue fever are found in this particular class. This entire hierarchy is shown in Fig. 2. Under “*disposition*” one also finds “*insecticide resistance*”. Although these terms were originally described in MIRO, the ontology for insecticide resistance [27], they have also been imported here in order to make it easier to be accessed by users, given the importance of vector control in the control of dengue fever. We should note here that “*insecticide resistance*” is considered to be a phenotype, and phenotypes are ontologically classified as being a “*quality*”. Such is the case, for example, in EFO, the Experimental Factor ontology [44] and the Ontology for General Medical Science, OGMS (https://code.google.com/p/ogms/). As a consequence, the Phenotypic Quality Ontology PATO (http://bioportal.bioontology.org/ontologies/PATO?p=summary) lists “*resistance to*” directly as “*quality*”. On the other hand, IDO [33], which we are using as a top-level ontology assigns resistance to “disposition”. We therefore follow IDO’s logic, but once the bio-ontology community solves this question, we will edit IDODEN as/if needed.

**Figure 3:**
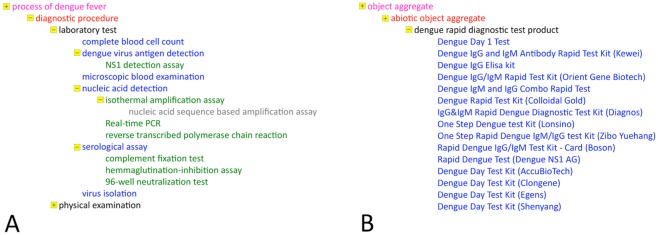
The classes “*process of dengue fever*” (A) and “*object* aggregate” (B) in IDODEN. Only terms relating to the diagnosis of dengue fever are shown. Font colours indicate the different levels of the hierarchy while a small yellow box in front of a term shows that it has children. The minus sign indicates that all child terms are listed while the plus sign indicates the presence of children terms that are not shown in the Figure.

A third example of completely practical nature follows. Diagnosis of dengue fever is usually performed clinically, especially in endemic countries; of course, it has to be confirmed through laboratory tests, particularly in order to differentiate it, in early stages, from other viral infections as well as to determine the virus’ serotype [2]. To this end several different diagnostic tests are being used to identify the virus, which, in IDODEN, can be found under the term “*diagnostic procedure*”, a child of the term “*process of dengue fever*” (Fig. 3A). Another method for the detection of the virus is the usage of ready-made test kits, which allows for an early confirmation of the diagnosis. The term for those kits used in IDODEN is “*dengue rapid diagnostic test product*“; the kits are listed in the “*object aggregate*” hierarchy as they are composed of more than one component (Fig. 3B). In IDODEN a kit only *participates_in* the process of virus detection which *results_in* the diagnosis of dengue.

**Figure 4:**
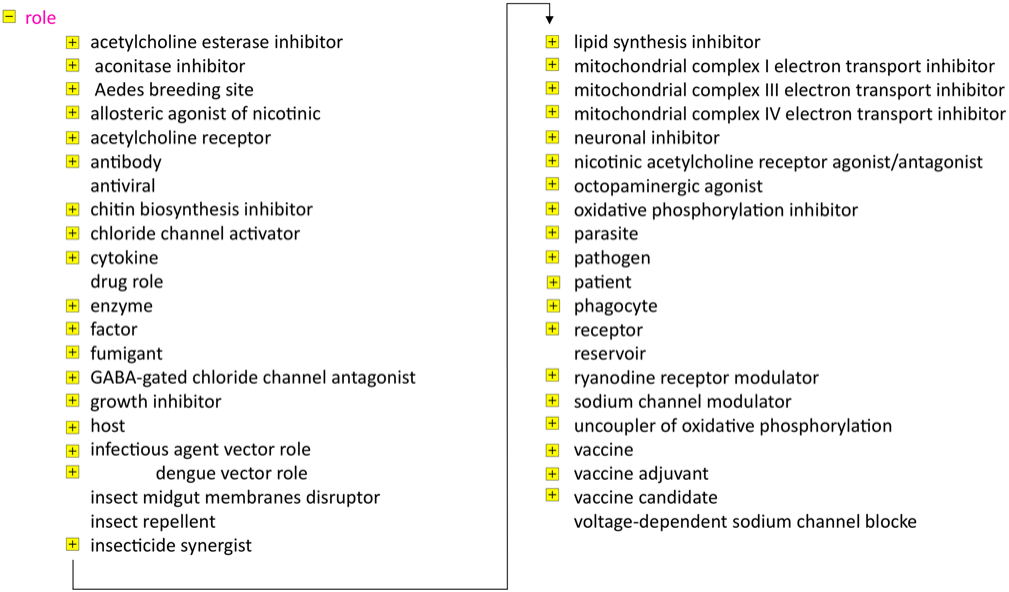
The complete class “*role*” in IDODEN. All terms shown are children of “role”. With the exception of the terms that are not preceded by a small yellow box all have children that are not shown. The arrow denotes that the terms in the right column follow those in the left one.

The next example we detail deals with the class “*role*”. This is one of the most populated classes of IDODEN. The “*role*” needs some additional description to better understand its significance. Its definition is “A realizable entity the manifestation of which brings about some result or end that is not essential to a continuant in virtue of the kind of thing that it is but that can be served or participated in by that kind of continuant in some kinds of natural, social or institutional contexts.” For example, we naturally tend to think of a mosquito as being a vector and dengue virus as being a pathogen (i.e. ontologically, *is_a* relationships);; this sounds correct to most ears, although this is ontologically incorrect. An instance of *Aedes aegypti* (i.e. a specific specimen) can very well exist in nature when it does not carry any dengue virus, thus if it actually does not act as a vector. A naive view would be to assign a second *is_a* relation to it, but since ontologies logically only allow for a single *is_a* relation for each entity, *Aedes aegypti* simply cannot be (thus no *is_a*) a vector. Rather, *Aedes aegypti* is a child of *Aedes*, which in turn is a descendant of “*biotic object*” (having gone through a simplified taxonomy). Similar ontologically correct relationships exist for a multitude of terms that are used in a jargon that is difficult to avoid even in scientific publications. The apparent problem is easily solved by assigning the relation *has_role* “*dengue vector*” to *Aedes aegypti* and the other species that transmit the viruses. Fig. 4 shows all roles listed in IDODEN. We stress here that among members of the ontology community there are several discussions as to the ontological “nature” of certain entities. Without going into long discussions, suffice to say that terms such “*enzyme*”, “*drug*”, “*parasite*” and many more are handled differently by others. Therefore, all candidate vaccines are *is_a* children of “*chemical compound*“; they all bear the role of “*candidate vaccine*”.

Finally, IDODEN also describes concepts for one of the main research interests of the dengue community, the development of a vaccine against the disease. Although, so far, no vaccine exists, clinical trials are already underway with different candidates in different phases [see 45, 46]. Given the significance of this endeavor we have incorporated in IDODEN all relevant and necessary terms related to vaccine development. In IDODEN the term “*dengue vaccine clinical trial*” is a child of “*process of dengue fever*”, the former term having the definition: “A processual entity by which a vaccine against dengue is tested clinically for safety and effectiveness”, while the latter is defined as “A process relating to dengue”. One hurdle in the development of a dengue vaccine is that the virus has four different serotypes (DENV-1–4) that have been known for longer, and a fifth (DENV-5) that is a recent discovery [40]. Thus, only vaccine candidates against an individual serotype (monovalent) or against the four serotypes known previously (tetravalent) exist. The way the ontology is structured allows for easy inclusion of new vaccine candidates against the DENV-5 when these are developed.

In IDODEN the children of the parent term “*VO:vaccine candidate*” have been classified in such a way as to reflect the different vaccine candidates in development. It has two children, “*vaccine candidate using part of organism*” and “*whole organism vaccine candidate*“. The former is a parent to “*DNA vaccine candidate*” and “*subunit vaccine candidate*”. Both of those terms are parents to children regarding their respective monovalent and tetravalent vaccine candidates. The term “*whole organism vaccine candidate*” has the subclass of “*live attenuated vaccine candidate*”, which is the parent of “*live attenuated viral vaccine candidate*”. The latter term has two subclasses, “*live attenuated monovalent dengue vaccine candidate*” and “*live attenuated tetravalent vaccine candidate*”.

**Figure 5:**
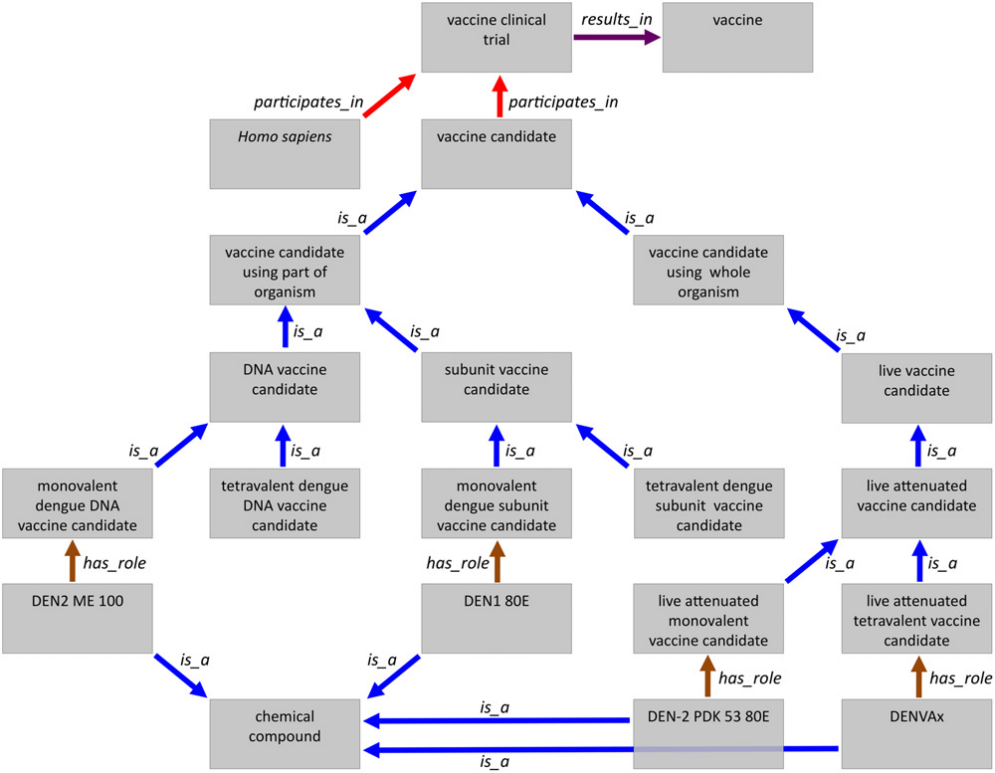
The class “*vaccine*” in IDODEN. Given the different relations linking the terms, we have used a non-hierarchical way of showing the vaccine calls and its children/related terms. The colours of the arrows indicate different relations.

The term “*VO:vaccine clinical trial*” which is a “*process of dengue fever*”, is of importance given the non-availability of vaccines. The above mentioned term “*VO:vaccine candidate*” *participate*s in a such a trial, as does “*Homo sapiens*“. The clinical trial can result in a “*vaccine*” which is an *agent_in* the “*IDOMAL:vaccination of population*” and “*vaccination against dengue*” (Fig. 5).

### IDODEN: Use in modeling

The first use of ontologies that comes to mind is that of the annotation of data for their inclusion in specialized databases. This stems from the huge success of the GO [11, 12] especially in the annotation of data derived from genome and transcriptome sequencing projects. This was one of the reasons that led VectorBase to the decision to construct ontologies that describe vector-borne diseases and related knowledge domains [25–28, 47]. Clearly, another area in which ontologies can be of great value, concerns overall standardization of data such that IT tools can interact (interoperability). Standardization is a *conditio sine qua non* for a most efficient use of DSSs. But in addition to these practical uses, ontologies can be excellent tools for modeling complex biological situations [9, 10]. A few examples will illustrate this for IDODEN. In all of them, terms are preceded by the primary ontology that described these entities in case this was not IDODEN.

**Figure 6:**
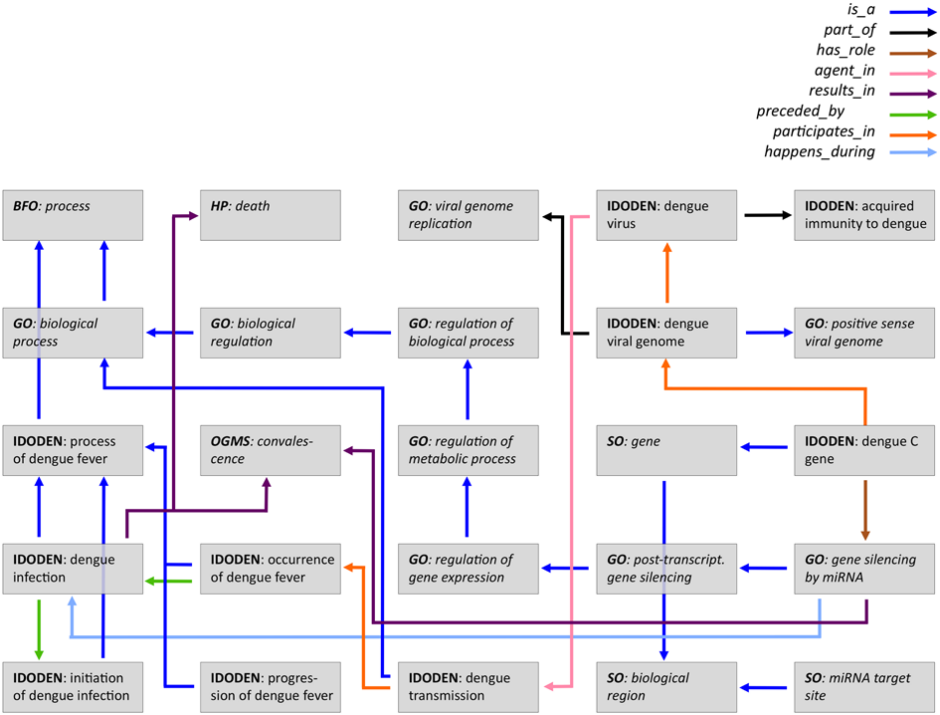
Ontological model of miRNA-mediated regulation of Dengue virus infection. The arrows show the different relations linking the ontology terms; the colours indicate different relations as listed above the model. Ontologies and terms in italics show classes that have been imported from ontologies other than IDODEN. Several terms have been omitted from the model.

Since dengue fever is caused by a virus and since it has been shown that *Aedes aegypti* mosquitoes, similar to *Drosophila melanogaster*, have the ability to silence gene expression [48], researchers have turned to RNA interference (RNAi) in an effort to suppress infection [49, 50], even creating artificial microRNAs to inhibit viral replication [51]. In IDODEN the infection, subsequent disease course, its outcome and the RNAi can be modelled as follows (Fig. 6). A “*dengue infection*” can result in two outcomes: i) “*HP:death*” or ii) “*OGMS:convalescence*”. The infection itself is preceded by the “*initiation of dengue infection*” which is part of the “*progression of dengue fever*”. The “*dengue infection*” itself is classified as a *process of dengue fever* which has the term “*BFO:process*” as its parent. In addition what happens during a potential “*dengue infection*” is that “*GO:gene silencing by miRNA*” is triggered which results in “*OGMS:convalescence*” [51]. This is classified as “*GO:posttranscriptional gene silencing*”, which is a “*GO:regulation of biological process*”, a child of “*GO:regulation of metabolic process*”, which is, in turn a “*GO:regulation of biological process*”, finally leading up to “*GO:biological regulation*”. Higher up this is a “*GO:biological process*” and the next step is the “*BFO:process*”. The “*dengue C gene*”, the gene responsible for the capsule of the virus [52], is connected with more than one relation to this mechanism. It participates in the “*GO:gene silencing by miRNA*”, while it has the role of a “*SO:miRNA target site*” and it is also a child of the term “*SO:gene*”. In our case “*SO:biological region*” is the parent term for both “*SO:miRNA target site*” as well as “*SO:gene*“. In addition to all of the above the “*dengue C gene*” is a *part_of* the “*dengue viral genome*” which is itself a *part_of* the “*dengue virus*”, which *participates* in an “*acquired immunity to dengue*”. The “*dengue virus*” is an *agent*_*in* the “*dengue transmission*” that participates in an “*occurrence of dengue fever*” which is *preceded_by* a “*dengue infection*”. We stress that this represents only a simplified model, in order to demonstrate the possibility of using IDODEN for modeling and therefore several terms/entities have been omitted.

**Figure 7:**
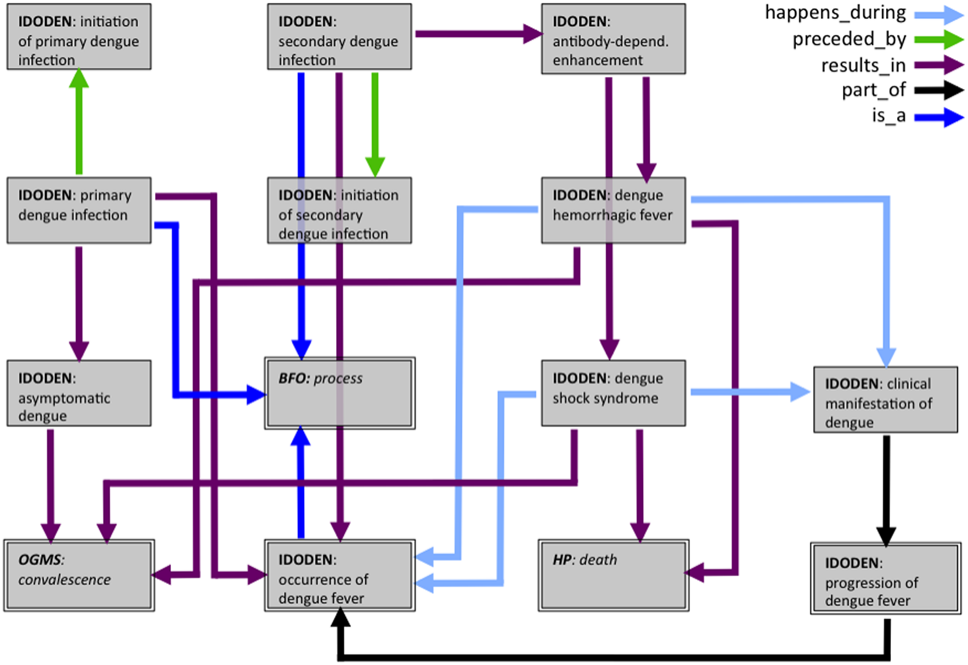
Ontological model of a dengue fever infection. The arrows show the different relations linking the ontology terms; the colours indicate different relations as listed at the upper right corner of the model. Ontologies and terms in italics show classes that have been imported from ontologies other than IDODEN. Several terms have been omitted from the model.

A second example illustrates the description of the course of the disease using IDODEN and other ontologies (Fig. 7). Dengue fever has two different forms, non-severe and severe, according to WHO; there are two severe forms, the dengue hemorrhagic fever and the dengue shock syndrome [1]. Most initial dengue infections result in the non-severe form of the disease [53]. The percentage of infected people increases when they suffer from a secondary infection from a different viral serotype [54]. Although there is no consensus as to what exactly causes this increase, it has been suggested that the antibody dependent enhancement (ADE) might be responsible [52, 55]. The “*primary dengue infection*” which is preceded by the “*initiation of primary dengue infection*” results in “*asymptomatic dengue*” which in the end results in “*OGMS:convalescence*”. According to the hypothesis of ADE a “*secondary dengue infection*”, which is preceded by the “*initiation of secondary dengue infection*” results in an “*occurrence of dengue fever”*. In addition, it results in the “*antibody dependent enhancement*” which can result in both “*dengue hemorrhagic fever*” and “*dengue shock syndrome”*. These severe forms can result in either “*OGMS:convalescence*” or in “*HP:death*”. In contrast to “*asymptomatic dengue”* the severe forms happen during an “*occurrence of dengue fever*” and the “*clinical manifestation of dengue*” which is part of the “*progression of dengue fever*”. This is itself a part of an “*occurrence of dengue fever*”. Interestingly, the infection model depicted here can be linked to the model of infection control through miRNAs. Five concepts are common to both models and one can join the two through those “crossroad” terms. This exemplifies how models can be expanded using either a comprehensive ontology and/or other ontologies that are built with interoperability in mind.

**Figure 8:**
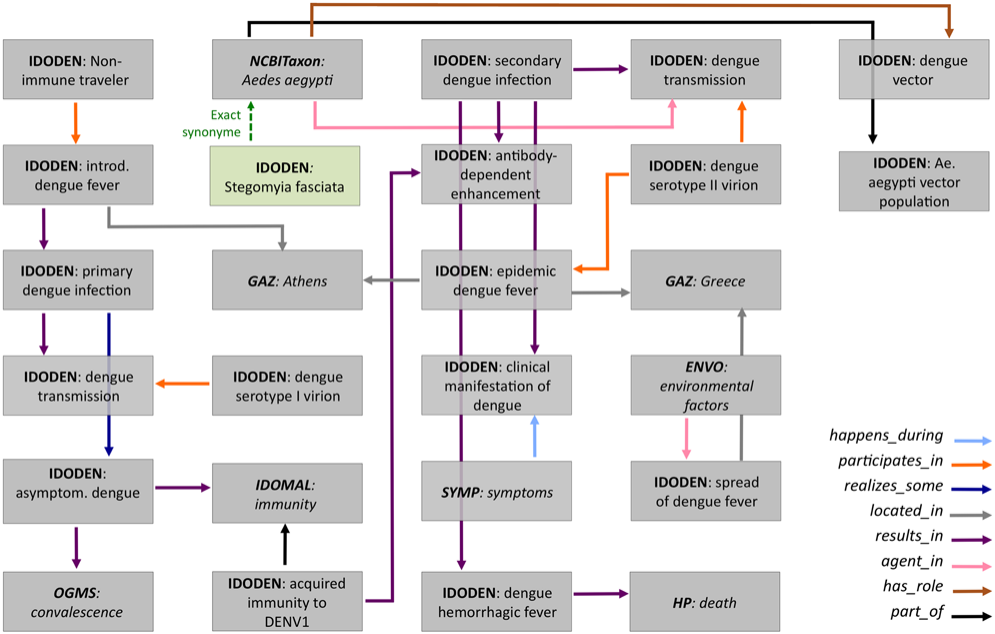
Ontological model of the 1927–1928 dengue fever epidemic in Athens. The arrows show the different relations linking the ontology terms; the colours indicate different relations as listed at the lower right corner of the model. Ontologies and terms in italics show classes that have been imported from ontologies other than IDODEN. The dashed green line denotes an “exact synonym relationship” between *Aedes aegypti* and *Stegomyia fasciata*, the term used for the former species in 1928. Several terms have been omitted from the model.

The last example of modeling based on ontologies deals with the course of an epidemic. We demonstrate this using the 1927–1928 dengue epidemic in Athens, Greece [56–60]. Fig. 8 shows how this epidemic, historically unique in several aspects, can be described ontologically. This was a biphasic epidemic that affected 693,000 patients (possibly 959,884), 1553 fatally (possibly 2700). The model that we present here also addresses the possibility of an initial DEN-1 infection followed by a DEN-2 infection, a highly possible fact, which has not yet been unequivocally resolved [57, 58]. Moreover, due to space constraints, several details are missing (*e.g.* the known vector of the infection, synonym *Stegomyia fasciata* for *A. aegypti*) as are all concepts that deal with the control measures taken at the time. Nevertheless, this model can be expanded almost *ad libitum* to cover all aspects of this historical event.

### Conclusions

Dengue fever is a debilitating disease. It was given the name “breakbone fever” due to the pain felt by the patients suffering from the disease. With the number of cases on a constant rise [1] and no therapy being available, emphasis was put in vaccine development. It was hypothesized that a vaccine could become reality in the next decade [61], although the newly discovered fifth serotype [40] could delay the development, given the fact that the now existing vaccine candidates only cover the four previously known ones.

With all that in mind, IDODEN was built in such a way that it covers all the important aspects for researchers and medical personnel, in order to assist their efforts. Furthermore, wherever possible, already existing terms from other ontologies were used in IDODEN. This is a common practice in ontology development and it was done in an attempt to maximize interoperability between already existing databases that rely on ontologies and to thus facilitate extensive searches on different aspects of the disease.

Many terms in the ontology have been included that cover ontologically entomological and epidemiological surveys that are underway. With those terms a database containing data of such surveys and using the ontology, will have a powerful search engine to quickly and easily get the relevant information, especially if combined with the recently developed ontology for vector surveillance and management [62].

The WHO-recommended diagnostic procedures and diagnostic tests are also included in IDODEN, thus enabling potential decision support systems to aid medical personnel in areas were dengue is endemic. It represents a focused way to access data and knowledge. The terms can be used to annotate data regarding outcomes of disease course and how effective those diagnostic procedures were at different stages of dengue fever. It could be even important for the comparison of the results of the diagnostic tests.

We have shown that IDODEN can indeed be used to model complex processes in such a way that they could be understood by machines (artificial intelligence) and humans alike, if the data are correctly annotated and the corresponding tools are able to be ontology-driven. IDODEN has the potential to drive databases, which are “smarter” than those that don’t utilize an ontology. This way, researchers will be able to identify the needs for their experiments more efficiently, e.g. being able to search for a specific dengue viral strain to see in which studies it was used and for what purposes. Such databases and other IT tools are not simply wishful thinking for the future, they are being built and used [18, 21], and IDODEN makes a substantial contribution to their growth and development.

We have shown here that IDODEN has the capacity to cover the different aspects of a complex disease. It will be a valuable tool in the research regarding dengue fever and it will be able to adapt to any new developments that may arise. If used correctly by the dengue community, IDODEN will prove to be an asset. Since it is built using the BFO and IDO on an upper level, cross-talk and integration with ontologies from other domains should be possible to compare or integrate the knowledge as needed. A closer collaboration between the different infectious disease ontologies should be a goal for the future.
